# Heterotrimeric G protein are involved in the regulation of multiple agronomic traits and stress tolerance in rice

**DOI:** 10.1186/s12870-020-2289-6

**Published:** 2020-02-28

**Authors:** Yue Cui, Nan Jiang, Zhengjin Xu, Quan Xu

**Affiliations:** 1 Rice Research Institute of Shenyang Agricultural University, Shenyang, 110866 China; 2 Shenyang Research and Development Service Center of Modern Agriculture, Shenyang, 110866 China

**Keywords:** Rice, Heterotrimeric G protein, CRISPR/Cas9, Yield components, Stress tolerance

## Abstract

**Background:**

The heterotrimeric G protein complex, consisting of Gα, Gβ, and Gγ subunits, are conserved signal transduction mechanism in eukaryotes. Recent molecular researches had demonstrated that G protein signaling participates in the regulation of yield related traits. However, the effects of G protein genes on yield components and stress tolerance are not well characterized.

**Results:**

In this study, we generated heterotrimeric G protein mutants in rice using CRISPR/Cas9 (Clustered Regularly Interspaced Short Palindromic Repeats) gene-editing technology. The effects of heterotrimeric G proteins on the regulation of yield components and stress tolerance were investigated. The mutants of *gs3* and *dep1* generated preferable agronomic traits compared to the wild-type, whereas the mutants of *rga1* showed an extreme dwarf phenotype, which led to a dramatic decrease in grain production. The mutants showed improved stress tolerance, especially under salinity treatment. We found four putative extra-large G proteins (PXLG)1–4 that also participate in the regulation of yield components and stress tolerance. A yeast two hybrid showed that the RGB1 might interact with PXLG2 but not with PXLG1, PXLG3 or PXLG4.

**Conclusion:**

These findings will not only improve our understanding of the repertoire of heterotrimeric G proteins in rice but also contribute to the application of heterotrimeric G proteins in rice breeding.

## Background

Much of the seminal work on heterotrimeric G proteins was accomplished over the past 40 years, and thus it is no wonder that this pathway is the best understood in the world. The heterotrimeric G proteins, comprising α, β, and γ subunits, perceive extracellular stimuli through cell surface receptors, and then transmit signals to effectors to initiate numerous cellular behaviors [1]. These include responses to hormones, drought, and pathogens, and developmental events such as lateral root formation, hypocotyl elongation, hook opening, leaf expansion, and silique development [2]. A large heterotrimeric G protein family exists in the mammalian genome, for example, the human genome encodes 23 Gα, 5 Gβ, and 12 Gγ [3, 4], whereas the heterotrimeric G protein repertoire in plants is much simpler than in animals [5]. In the rice genome, there are single-copy of canonical Gα (*RGA1*) and Gβ (*RGB1*) subunits [6, 7], and two canonical Gγ subunits (*RGG1* and *RGG2*) [8]. Recent molecular studies have found that there are three unusually large proteins with sequences similar to Gα subunits in the genome of *Arabidopsis*, these are named extra-large GTP-binding proteins, or XLGs [9]. The *XLGs* have a long cys-rich domain in the N-terminal compared to the canonical Gα, and all three *XLGs* of the Gα subunit show GTP-binding and GTPase activity [10, 11]. Similarly, there are three non-canonical Gγ subunits, *GS3*, *DEP1*, and *GGC2* in the rice genome [12]. The non-canonical Gγ subunits also have a long cys-rich domain in the C-terminal compared to *RGG1* and *RGG2* [13–15]. Interestingly, the variation in the cys-rich domain contributes to the improvement of important agronomic traits. The varieties harboring different *GS3* alleles generated diversity in grain sizes [16, 17], and a deletion of the cys-rich domain of the *dep1–1* allele resulted in a dense and erect panicle architecture, and a significant increase of grain number per panicle [18–20]. Taken together, the heterotrimeric XLG proteins and non-canonical Gγ subunits that do not exist in the repertoire of heterotrimeric G proteins in animals might identify additional signal transduction pathways and expand the repertoire of heterotrimeric G proteins.

CRISPR/Cas9 systems have been successfully applied as efficient genome editing tools in a number of plant species [21–24]. The CRISPR/Cas9 gene-editing technology has been used to verify the function of important yield-related genes, such as *DEP1*, *GS3*, *IPA1*, and *Gn1a* [22]. In the present study, we generated one Gα mutant, one Gβ mutant, five Gγ mutants, and four extra-large Gα mutants using CRISPR/Cas9 gene-editing technology. We assessed the yield components and stress tolerance of the mutants, revealing the distinctly different effects of the heterotrimeric G protein mutants on yield components and stress tolerance. We aimed to gain insight into the heterotrimeric G proteins in the rice genome, with the goal of providing better information and germplasm resources for rice breeding.

## Results

### Heterotrimeric G protein-encoding gene investigation of the transformation receipt

The *japonica* rice variety Sasanishiki was used as wild type (WT) for the transformation in this study. Sasanishiki is a typical Japanese *japonica* variety that was widely cultivated in Northeast China before the 1980s. Sasanishiki then became a major backbone parent, and many rice varieties in Northeast China share the genome segment from Sasanishiki. Sasanishiki thus represents an ideal receipt for transformation in this study, not only for the functional demonstration of heterotrimeric G protein-encoding genes in rice, but also for the genetic improvement by molecular breeding in Northeast China. Thus, we first assessed the heterotrimeric G protein-encoding genes of Sasanishiki. The Gα, Gβ, and five Gγ genes of Sasanishiki were investigated (Table 1). The results showed that Sasanishiki shared the G protein genes to Nipponbare. No frame shift was detected between Nipponbare and Sasanishiki. Thus, we assumed that all seven G protein genes in Sasanishiki were functional.

**Table 1:** Details of the target genes modified in this study

Heterotrimeric G protein	Gene	Locus	Function	Reference
Gα	RGA1	Os05g0333200	F	ángel Ferrero-Serrano et al. (2016, 2018) [25, 26]
Gβ	RGB1	Os03g0669200	F	Zhang et al. (2015) [7]; Utsunomiya et al. (2011) [27]
Gγ	RGG1	Os03g0635100	F	Xu et al. (2016) [28]
	RGG2	Os02g0137800	F	Miao et al. (2019) [29]
	GS3	Os03g0407400	F	Mao et al. (2010) [30]
	GGC2	Os08g0456600	F	Xu et al. (2016) [28]
	DEP1	Os09g0441900	F	Huang et al. (2009) [18]; Wang et al. (2009) [20]

F represents functional alleles

### Generation of heterotrimeric G protein mutants using CRISPR/Cas9

**Fig. 1:**
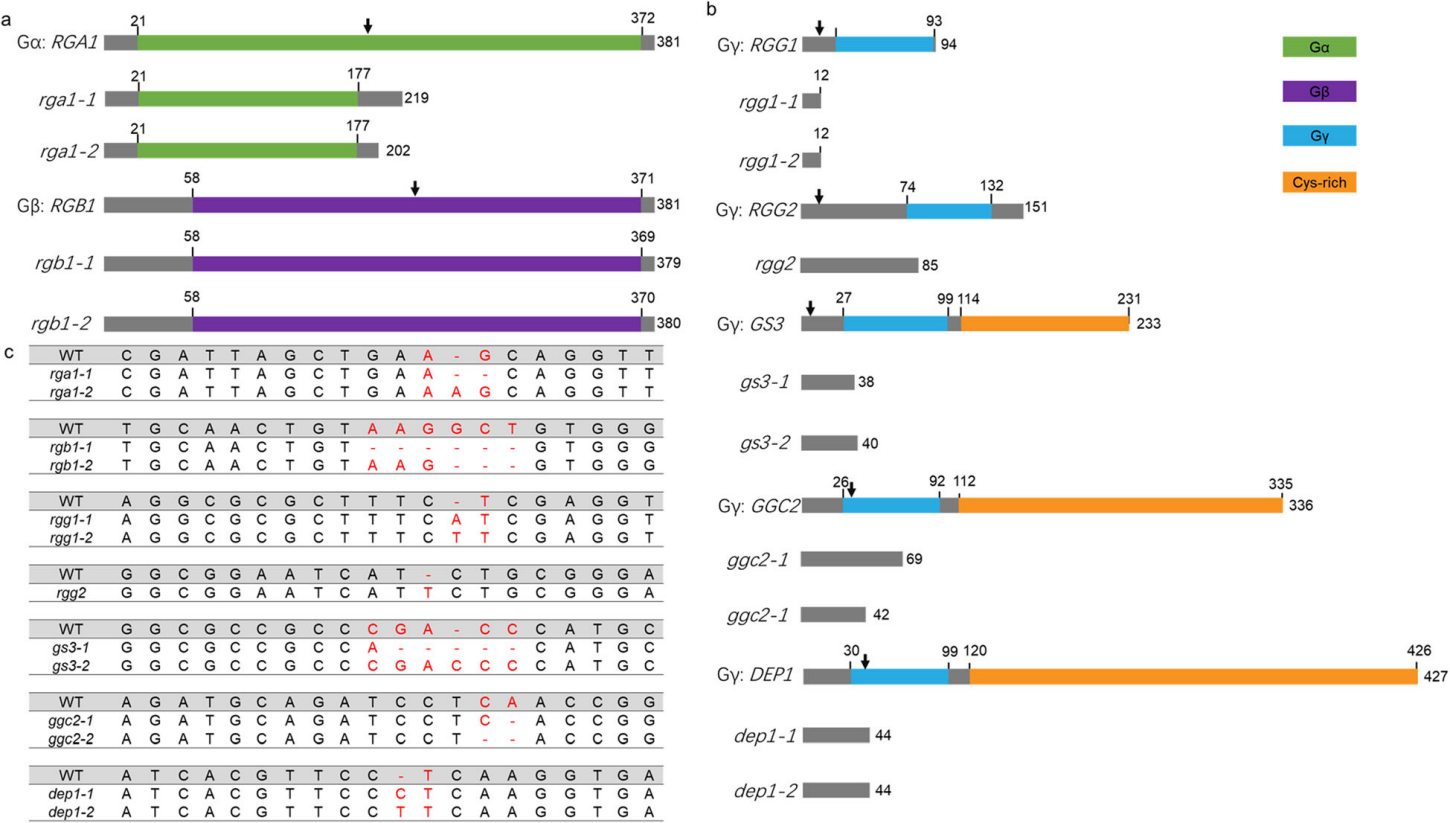
The schematic representation of the heterotrimeric G proteins and the sequences of the mutants generated by CRISPR/Cas9 gene editing. **a** The schematic representation of the heterotrimeric Gα and Gβ proteins in rice. The functional domains are shown in different colors. The black arrow indicates the position of the sgRNA. The number indicates the amino acid sequence. **b** The protein schematic representation of the heterotrimeric Gγ proteins in rice. The functional domains are shown in different colors. The black arrow indicates the position of the sgRNA. The number indicates the amino acid sequence. **c** The sequences of the heterotrimeric G protein mutants generated by CRISPR/Cas9 gene editing

**Fig. 2:**
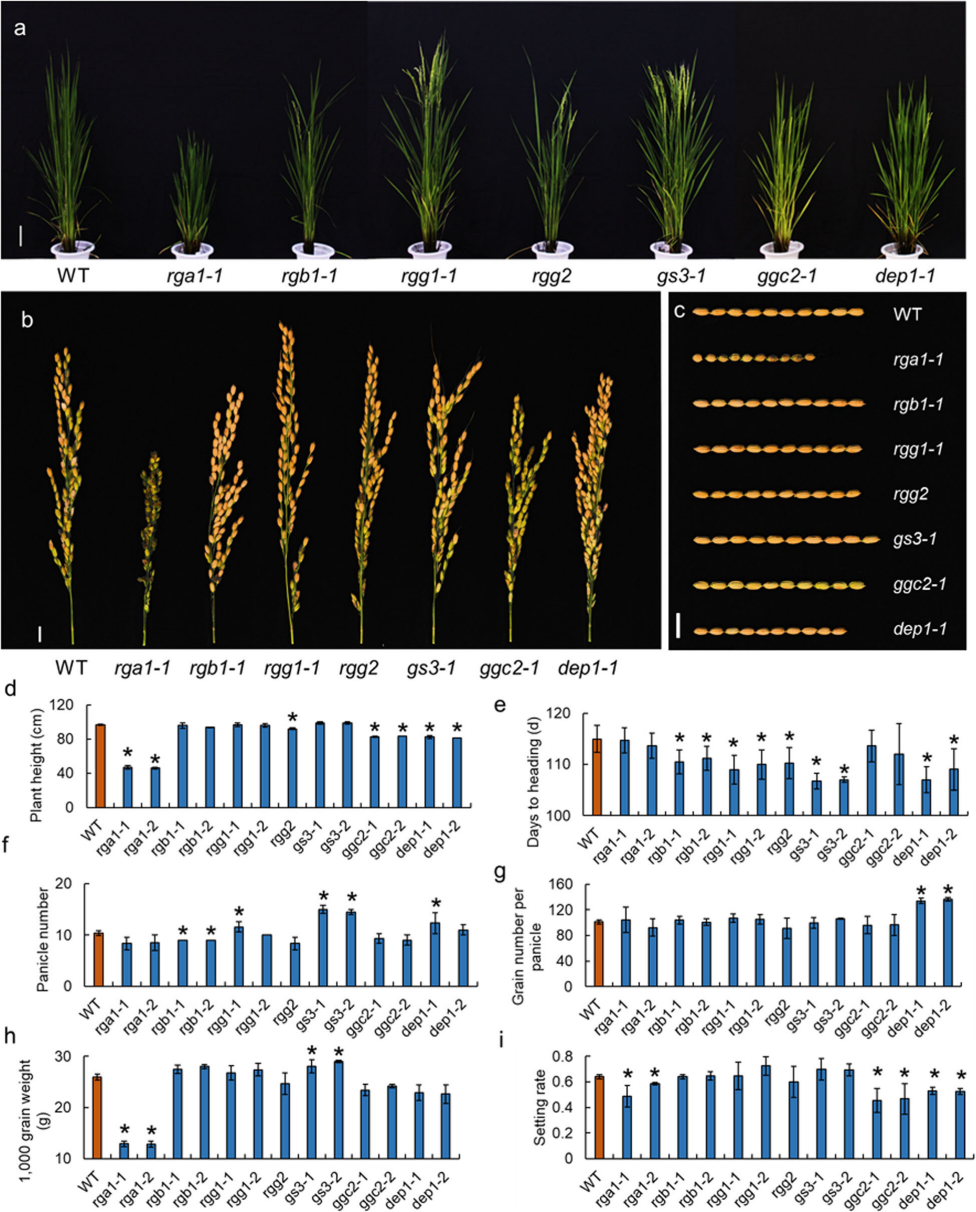
Genetic effects of the heterotrimeric G protein on plant architecture, panicle size, grain size, and yield components. **a** The whole plants of the heterotrimeric G protein mutants. Bar = 10 cm (**b**) The panicle of the heterotrimeric G protein mutants. Bar = 1 cm (**c**) The grains of the heterotrimeric G protein mutants. Bar = 1 cm (**d**–**i**) The plant heights, heading times, panicle numbers, grain numbers per panicle, 1000-grain weight, and setting rates of the heterotrimeric G protein mutants. * indicates significance at the 5% level

In order to evaluate the effects of heterotrimeric G protein-encoding genes on rice agronomic traits, we used CRISPR/Cas9 to specifically induce mutagenesis of the G protein. We sequenced 20 plants of each mutant to examine the mutation efficiency in the T_0_ generation. The result showed that mutations occurred in 64.29% of plants, and 10.71% of sequenced plants had a putative homozygous mutation (Fig. 1 and Table 2). Seven T_1_ lines with homozygous mutations and the WT were used in further analyses. At least two independent transgenic lines were obtained for each Heterotrimeric G protein encoding gene except for *rgg2* (Fig. 1). Forty T_1_ plants for each mutant and WT were sown in the experimental field of Shenyang Agricultural University (N41°, E123°) on 23 April 2018. We recorded the heading time when the first panicle had emerged from the sheath. The results showed that the *rgb1*, *rgg1*, *rgg2*, *gs3,* and *dep1* mutants underwent heading significantly earlier than the WT. The *rga1* and *ggc2* mutants showed a similar heading time as the WT (Fig. 2).

**Table 2:** Percentage of T0 plants found with mutations in target gene

Target gene	No. of plants examined	Plants with mutations		Putative homozugous mutations	
		Number	Mutation rate (%)	Number	Mutation rate (%)
RGA1	20	15	75.00	1	5.00
RGB1	20	13	65.00	0	0.00
RGG1	20	13	65.00	7	35.00
RGG2	20	14	70.00	4	20.00
GS3	20	18	90.00	1	5.00
GGC2	20	3	15.00	0	0.00
DEP1	20	14	70.00	2	10.00
Total	140	90	64.29	15	10.71

### The yield components of heterotrimeric G protein mutants

The heterotrimeric G protein is involved in the regulation of multiple agronomic traits, including grain size and panicle architecture. Thus, we compared the yield components of the seven G protein mutants and the WT (Fig. 2). The results showed that the independent mutant lines exhibited similar phenotypic changes. The *rga1* showed a significant dwarf phenotype, with a dramatic decrease in panicle length, grain length, 1000 grain weight, and setting rate compared to that of the WT. The *rgb1* mutant exhibited a semi-dwarf phenotype, and the panicle number was less than that of the WT. Sun et al. and Gao et al. reported that the null mutant of *RGB1* generated by CRISPR/Cas9 gene editing technology was lethal [31, 32]. However, the three base and six base deletions in *rgb1–1* and *rgb1–2* did not cause a frame shift, which might be the reason for the survival of *rgb1–1* and *rgb1–2*. In the Gγ subunits, the *rgg1* mutant had longer but fewer panicles than that of the WT, and the *rgg2* mutant showed a decrease in plant height compared to that of the WT. The *ggc2* and *dep1* mutants exhibited a decrease in plant height, and the *dep1* mutant had a shorter grain but a greater grain number per panicle compared to that of the WT. The *gs3* mutant had a higher panicle number per plant and a longer grain length, which led to an increase in the 1000 grain weight compared to that of the WT.

### The stress tolerance of the heterotrimeric G protein mutants

**Fig. 3:**
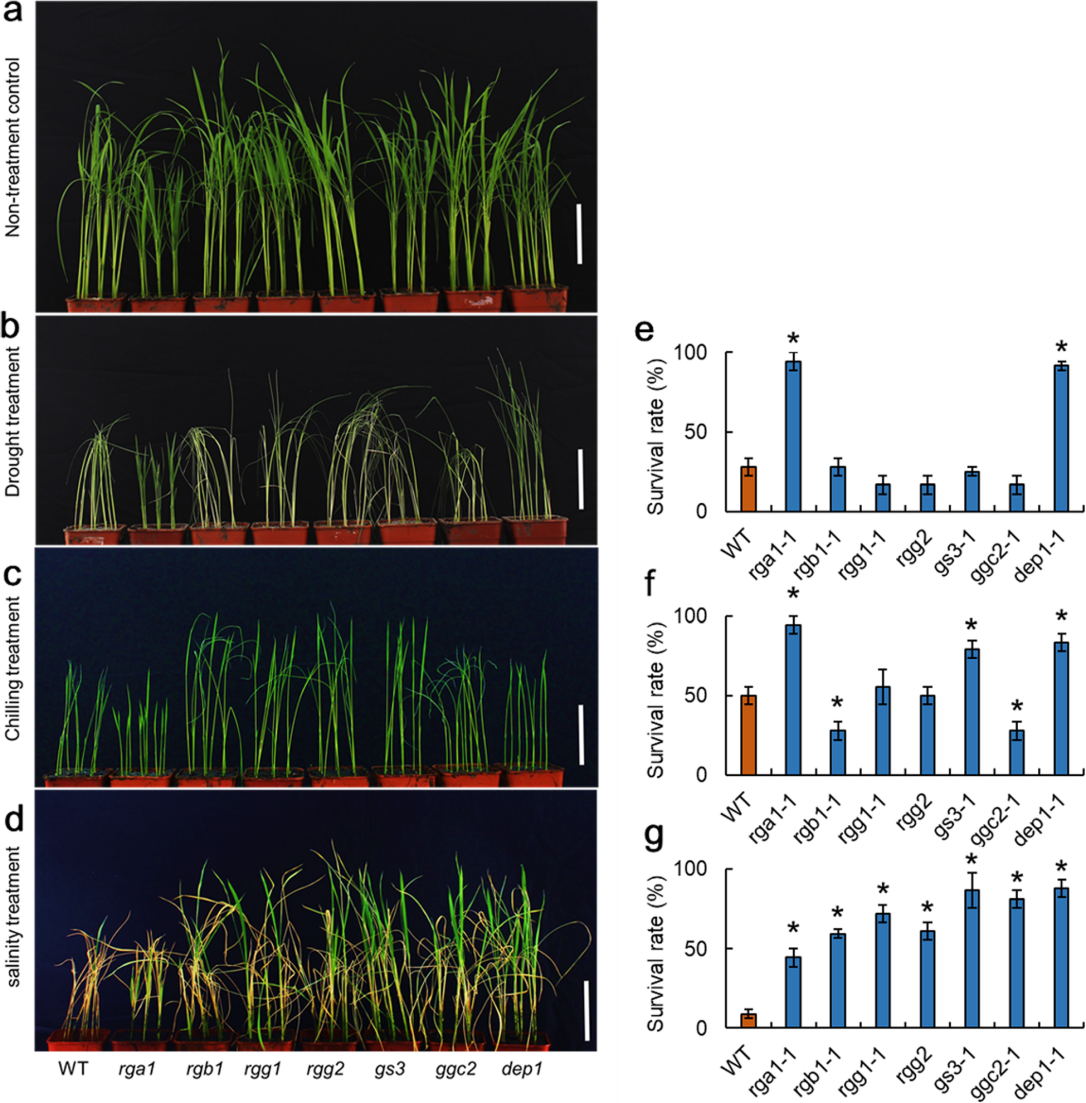
The stress tolerance of the heterotrimeric G protein mutants. **a** The non-treatment control of WT and heterotrimeric G protein mutants. **b**–**d** Photos of the plants treated with drought, chilling, and salinity stress. **e**–**g** The survival rates after drought, chilling, and salinity treatment. Bar = 10 cm, * indicates significance at the 5% level

We then examined the drought, chilling, and salinity tolerance of the heterotrimeric G protein mutants (Fig. 3). As the independent mutant lines exhibited similar phenotypic changes, we only used the number 1 transgenic line for each heterotrimeric G protein for the stress tolerance survey. In the drought tolerance survey, the *rga1* and *dep1* mutants showed an enhanced drought tolerance compared to the WT, whereas the *rgb1*, *rgg1*, *rgg2*, *gs3*, and *ggc2* mutants exhibited similar deficiencies caused by drought. In the chilling treatment test, the *rgb1* and *ggc2* mutants were more sensitive than the WT. The *rga1* and *dep1* mutants showed improved chilling tolerance compared to the WT. In the salinity treatments, all of the heterotrimeric mutants showed enhanced salinity tolerance compared to the WT, especially the *gs3* and *dep1* mutants. Taken together, these results suggest that the heterotrimeric G protein might participate in multiple stress response mechanisms.

### The extra-large Gα protein in rice

**Fig. 4:**
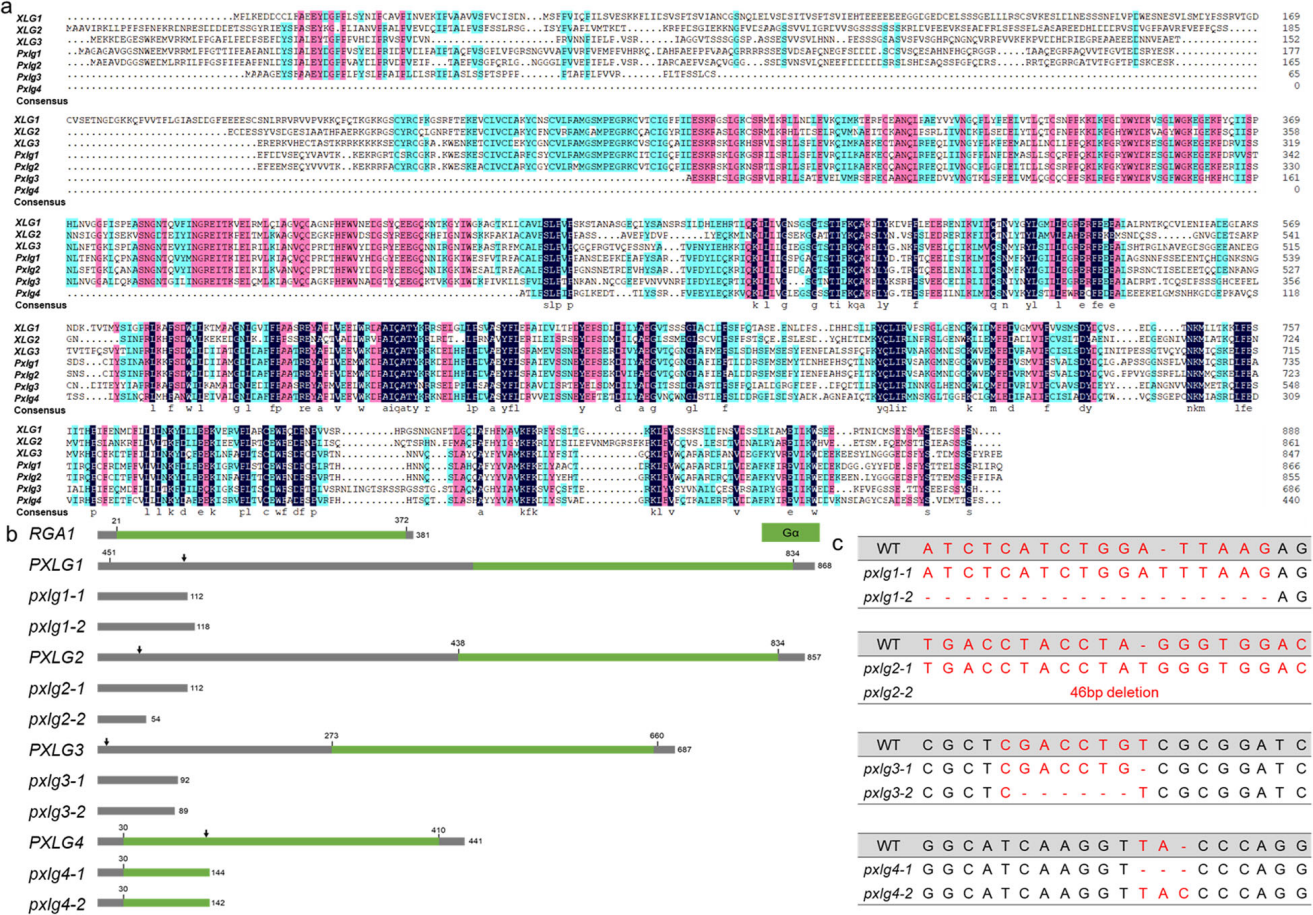
The putative extra-large Gα protein in rice. **a** A phylogenetic analysis of the extra-large G protein in *Arabidopsis* and rice, in which identical and conserved residues are indicated by different colors. **b** The schematic representation of the putative extra-large Gα protein in rice. The black arrow indicates the position of the sgRNA. **c** The sequence of the putative extra-large Gα protein mutant generated by CRISPR/Cas9 gene editing

**Fig. 5:**
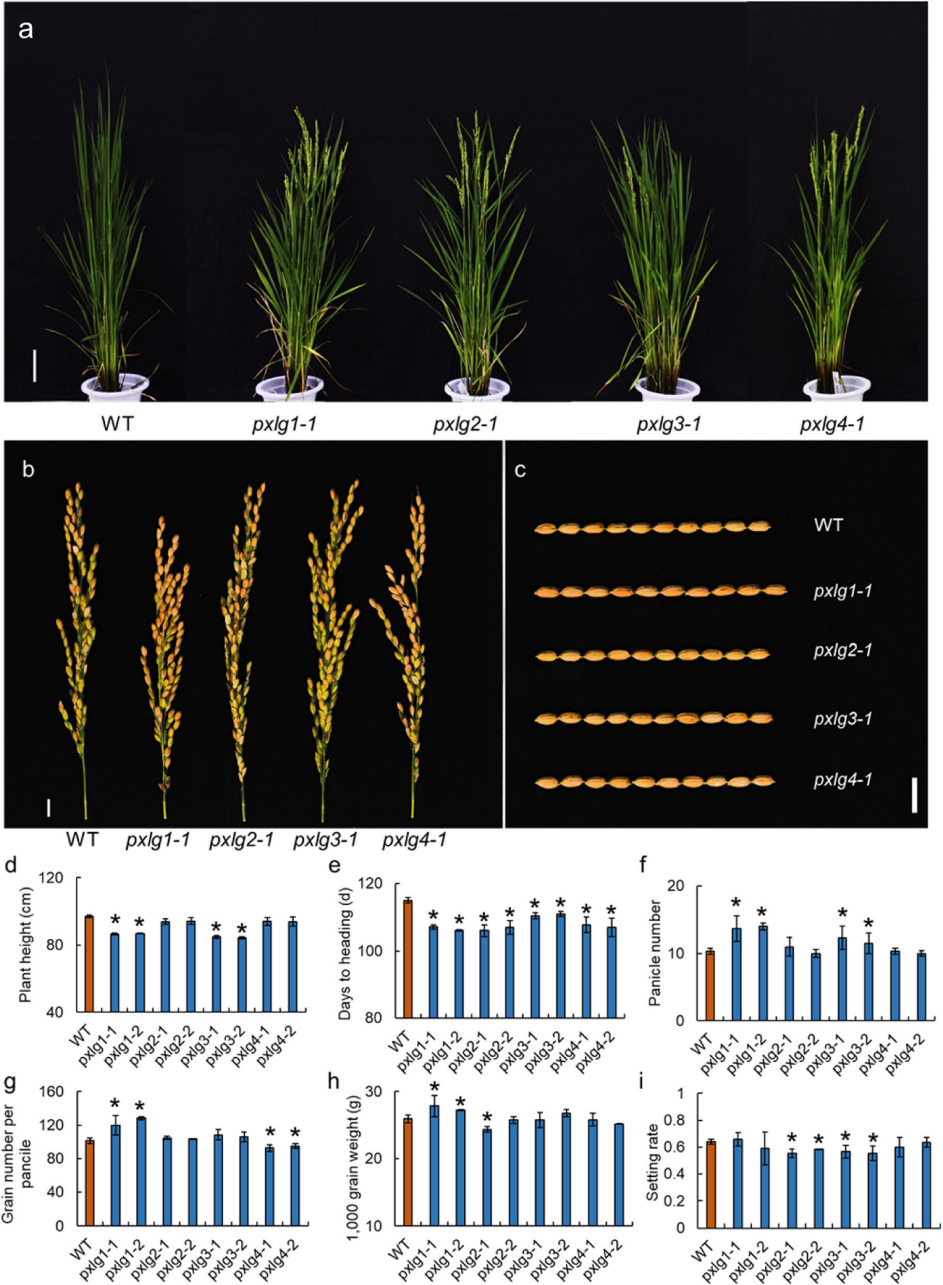
Genetic effects of the putative extra-large Gα protein on plant architecture, panicle size, grain size, and yield components. **a** The whole plants of the putative extra-large Gα protein mutants. Bar = 10 cm. **b** The panicles of the putative extra-large Gα protein mutants. Bar = 1 cm. **c** The grains of the putative extra-large Gα protein mutants. Bar = 1 cm. **d**–**i** The plant heights, heading times, panicle numbers, grain numbers per panicle, 1000-grain weight, and setting rates of the putative extra-large Gα protein mutants. * indicates significance at the 5% level

**Fig. 6:**
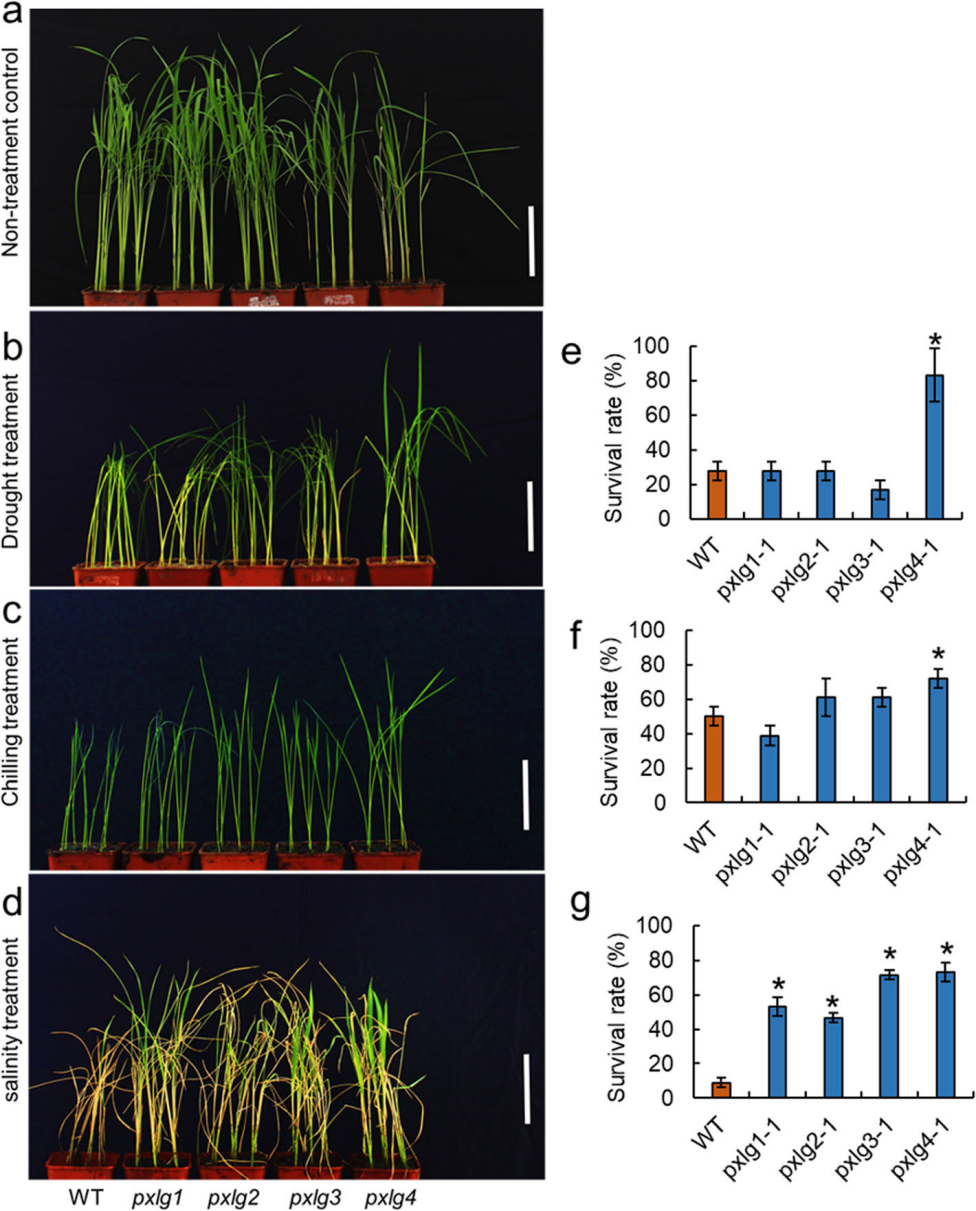
The stress tolerance of the putative extra-large Gα protein mutants. **a** The non-treatment control of the WT and putative extra-large Gα protein mutants. **b**–**d** Photos of plants treated with drought, chilling, and salinity stress. **e**–**g** The survival rates after drought, chilling, and salinity stress. Bar = 10 cm. * indicates significance at the 5% level

**Fig. 7:**
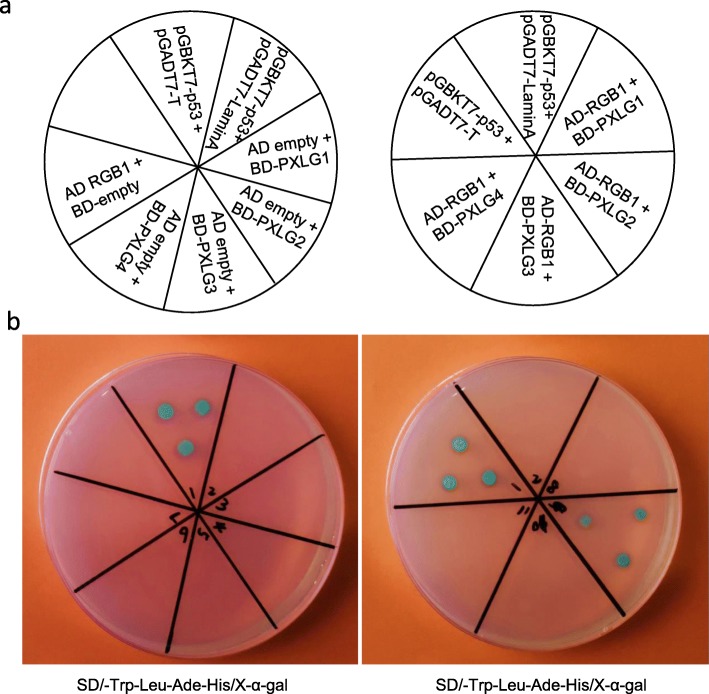
Interaction of RGB1 with PXLGs. In the yeast two hybrid assay, RGB1 is used as a prey (AD), and the PXLGs are used as bait (BD) (**a**) The corresponding positions in the self-activation test and yeast two hybrid assay. **b** The result of self-activation test and yeast two hybrid assay. Two plasmids containing either an AD or BD construct were introduced into a yeast strain and transformants were grown on selective medium lacking Leu and Trp. pGBKT7-p53 + pGADT7-T was used as a positive control, and pGBKT7-p53 + pGADT7-LaminA was used as a negative control

The bioinformatics analysis showed that there were four putative extra-large Gα protein (*pxlg1–4*) encoding genes in the genome of rice (Table 3). We then generated mutants of four *pxlg* genes (Fig. 4). The four *pxlg* mutants showed an early flowering phenotype compared to the WT (Fig. 5). The *plxg1* mutant had a shorter panicle but a longer grain than that of the WT. The panicle number of *pxlg1* was significantly increased compared to that of the WT. The settings rate of *pxlg2* and *pxlg3* were decreased compared to that of the WT. The *pxlg1* had a longer grain length than that of the WT, that led to an increase in 1000 grain weight. The two independent transgenic lines of *pxlg2* showed slight differences in 1000 grain weight, the *pxlg2–1* showed significantly decrease in 1000 grain weight, whereas the *pxlg2–2* had a similar 1000 grain weight compared to the WT. We also investigated the stress tolerance of the four *pxlg* mutants (Fig. 6). In the drought treatment, *pxlg4* showed enhanced drought tolerance compared to the WT and the other three *pxlgs*. In the chilling treatment, *pxlg4* exhibited improved chilling tolerance compared to the WT. In the salinity treatment, all four *pxlgs* showed enhanced salinity tolerance, particularly *pxlg3* and *pxlg4,* which had greater chilling tolerance than that of WT, *pxlg1,* and *pxlg2*. We subsequently surveyed whether the PXLGs interacted with RGB1 in rice using a yeast two hybrid analysis. The results showed that RGB1 interacted with PXLG2, but not with PXLG1, PXLG3, or PXLG4 (Fig. 7).

**Table 3:** Percentage of T0 plants found with mutations in target gene

Putative extra-large G protein	Locus	Function	No. of plants examined	Plants with mutations		Putative homozugous mutations	
				Number	Mutation rate (%)	Number	Mutation rate (%)
pxlg1	Os06g0111400	F	20	12	60.00	4	20.00
pxlg2	Os11g0206700	F	20	11	55.00	2	10.00
pxlg3	Os12g0593000	F	20	10	50.00	1	5.00
pxlg4	Os10g0117800	F	20	11	55.00	1	5.00
Total			80	44	55.00	8	10.00

F represents functional alleles

## Discussion

The heterotrimeric G proteins participate in a number of biological processes. Previous studies have shown that heterotrimeric G protein mutants exhibited various phenotypic changes. The null mutant of the *RGA1* gene exhibits a severe dwarf phenotype in rice [33, 34], whereas the down-regulation of the expression of the *RGA1* gene causes a semi-dwarf phenotype [19]. Microarray analysis of *RGA1* showed that *RGA1* might participate in the regulation of multiple abiotic stresses, such as drought, salinity, heat, and cold tolerance [35]. During drought, *rga1* mutant plants exhibit greater stomatal conductance than the WT, but both genotypes exhibit the same transpirational water loss per unit leaf area [25]. A recent study showed that *RGA1* could regulate photoprotection and photoavoidance in rice [26]. The *COLD1* gene could regulate Gα to confer chilling tolerance in rice [36]. Our studies confirmed that the null mutants of *RGA1* showed a severe dwarf phenotype and exhibited stress tolerance under drought, chilling, and salinity treatments. The suppression of *RGB1* causes dwarfism and browning of the internodes and lamina joint regions [27], and the null mutant of *RGB1* was lethal under the genetic background of ZH11 [31, 32]. Our study also showed that the mutation of *rgb1* caused a dwarfish plant architecture, with the panicle length being shorter than that of the WT. We concluded that *rgb1–1* and *rgb1–2* harbor mild alleles of *RGB1* as the 3-bp and 6-bp deletions did not cause frame shifts. Overexpression of *RGG2* in Nipponbare led to a reduced plant height and decreased grain size, whereas the mutants generated via CRISPR/Cas9 in the Zhenshan 97 background exhibited enhanced growth, including elongated internodes, increased 1000-grain weight and plant biomass, and enhanced grain yield per plant [29]. However, the *rgg2* CRISPR/Cas9 gene-edited plants under the Sasanishiki genetic background showed a similar phenotype to that of WT (Fig. 2). Interestingly, the mutant of *rgg1* showed enhanced growth, which was similar to the *rgg2* mutants in the Zhenshan 97 genetic background (Fig. 2). These results demonstrate that both *RGG1* and *RGG2* might act as regulators of plant growth in rice. Recent molecular studies identified two non-canonical Gγ subunits *DEP1* and *GS3* as major quantitative trait loci (QTLs) [28]. Several studies identified the *GS3* locus as a major QTL for grain length, grain width, and grain weight, and *DEP1* corresponded to the dense erect panicle and increased grain number [18, 30, 37]. Our study showed that the knock-out mutation of *gs3* could significantly increase the grain length compared to that of the WT, whereas the knock-out mutant of *dep1* showed significantly decreased grain length. The *dep1* mutants had a shorter panicle than the WT but exhibited a significant increase in grain number per panicle. The mutants of another atypical Gγ protein gene, *GGC2*, did not exhibit a preferential phenotype as observed in the *gs3* and *dep1* mutants. The panicle size and setting rate were penalized in the *ggc2* mutant. Our study also indicated that the *rgb1*, *rgg1*, *rgg2*, *gs3* and *dep1* mutants underwent heading significantly earlier than the WT, results that had not been reported in previous studies. The early heading phenotype observed in the present study indicated that the G protein signaling might participate in the control network of plant heading.

Recently, a “self-inhibition” model was constructed to explain the gain of function mutants of *dep1* and *gs3*. The hypothesis is that the C-terminal domains inhibited the N-terminal domains in non-canonical Gγ proteins [12, 30]. Although both C-terminal and N-terminal regions is thought to be involved in protein interaction, the molecular mechanisms of the observed self-inhibition of *GS3* and *DEP1* remain elusive [19, 30]. As *DEP1* plays a crucial role in the understanding of G protein in rice and in rice breeding, a number of studies employing CRISPR/Cas9 technology have been conducted to verified the function of *DEP1* [22, 31, 38–41]. The *DEP1* CRISPR/Cas9 gene-edited plants with an eliminated C-terminal exhibited an increase in grain number per panicle, reductions in plant height, panicle length, and grain length, and an erect panicle architecture, whereas the plants that had lost both the Gγ and cys-rich domains showed reductions in setting rate and grain number per panicle. Additionally, we noticed the truncated *dep1* with an eliminated C-terminal also exhibited a reduction in grain number per panicle under the genetic background of Nongken 57 and Wuyunjing 8 [42, 43]. These results suggested the truncated *dep1* alleles could generate opposite effects to grain number per panicle under different genetic background. The present study showed that the mutants with both the Gγ domain and cys-rich domains eliminated showed an increase in grain number per panicle (Fig. 2). Li et al. generated three lines harboring different length of truncation at C-terminal of *DEP1* using CRISPR/Cas9 gene editing technology, and the three lines exhibited similar phenotypic changes of the grain size [40]. Taken together, we hypothesized that both the two types of *DEP1* alleles (the allele eliminated Gγ and cys-rich domains, and allele eliminated only cys-rich domains) could increase the grain number per panicle, the opposite effects might due to the different genetic background, and a complex genetic network might exist in heterotrimeric G protein signaling in rice.

Studies of the extra-large Gα protein in *Arabidopsis* have demonstrated that the heterotrimeric XLG-Gβγ proteins represent additional signal transduction mechanisms in plant heterotrimeric G protein signaling [9–11, 44]. However, the function of *XLGs* in the rice genome is unclear. The bioinformatics analysis indicated that there were four putative extra-large Gα protein encoding genes (*PXLG1–4*) in the genome of rice. We generated the mutants of four *pxlg1–4* mutants using CRISPR/Cas9. All four *pxlg* mutants showed an earlier heading phenotype than the WT, and various phenotypic changes in yield components and stress tolerance were observed in the comparison between the WT and mutants. As the yeast two hybrid analysis showed that RGB1 could only interact with PXLG2, we expected that the mutant of *pxlg2* could generated obvious agronomic changes compared to other mutants of *PXLGs*. However, the mutant of *pxlg2* showed mediocre phenotypic change among the four mutants of *pxlgs*, whereas the mutant of *pxlg1* showed more severe changes in panicle length and grain size, and mutant of *pxlg4* showed improved stress tolerance compared to other mutants of *PXLGs*. These results indicated that PXLG2 might participate in the regulation of yield components and tress tolerance through the direct interaction with RGB1. Nevertheless, the interaction among four PXLGs needs further study.

Yield is the latest addition to the growing list of critical traits modulated by heterotrimeric G signaling in plants [12]. By combining different G protein variants, the grain length could be decreased by up to 35% or increased by up to 19%, leading to an over 40% decrease or a 28% increase in grain weight [31]. The present study constructed heterotrimeric G protein mutants in an identical genetic background. The effects of the heterotrimeric G protein in the regulation of yield components and stress tolerance were assessed. We found that the *gs3* and *dep1* mutants showed an ideal phenotype regarding both yield components and stress tolerance. The *pxlg4* mutant exhibited similar yield components but enhanced stress tolerance compared to the WT. The grain yield per plant of the WT was 17.38 ± 0.99 g, and those of *pxlg1–1* and *pxlg1–2* were 29.96 ± 1.59 g and 28.88 ± 0.95 g, respectively. Thus, *pxlg1* could improve the grain yield per plant by 69% under our experimental conditions, indicating that PXLG1 has a potential application in high yield rice breeding. Taken together, the results from the manipulation of G protein signaling might lead to improvements in yield and stress tolerance in rice and other crop species.

## Conclusions

The present study generated one Gα, one Gβ, and five Gγ mutants using CRISPR/Cas9 gene-editing technology. The bioinformatics analysis showed that there were four putative extra-large Gα protein encoding genes (*PXLG1–4*) in the genome of rice. The yield components and stress tolerance were surveyed. The results showed that the heterotrimeric G proteins were involved in the regulation of yield components and stress tolerance. The yeast two hybrid assay showed that PXLG2 might interact with RGB1. These findings not only improve our understanding of the repertoire of heterotrimeric G proteins in rice but also contribute to the application of heterotrimeric G proteins in rice breeding.

## Methods

### CRISPR/Cas9 vector construction and plant transformation

The experiment was conducted under the genetic background of the Japanese commercial *japonica* cultivar Sasanishiki. The seed of Sasanishiki (JP number: 5354) was ordered from the Genebank Project, NARO (Tsukuba, Japan). According to the standard material transfer agreement of Genebank, the seeds from Genebank were available to conduct scientific research and education. In order to perform the CRISPR/Cas9 gene editing, we conducted the vector construction as described in our previous study [45]. We conducted the rice transformation according to previously described method [46]. Genomic DNA was extracted from the mutant plants, and primer pairs flanking the designed target site were used for PCR amplification. The 200–500 bp PCR products were sequenced directly using the Degenerate Sequence Decoding method [21]. The primer used in the present study was listed in additional file 1.

### Field experiments

Field experiments were conducted at the experimental farm of Shenyang Agricultural University, Shenyang, China (N41°, E123°) in 2018. Two independent transgenic lines for each heterotrimeric G protein were prepared except for *rgg2*, which only had single transgenic line. Seeds of mutants and WT were sown on 24 April, and transplanting (one seedling per hill) was conducted on 23 May. A randomized block design with three replicates was used in the present study. The plot was 5.4 m^2^ and contained 120 plants with 30 cm × 15 cm intervals. The cultivation method and field management were as described previously [38]. We harvested the aboveground parts of 24 plants for each transgenic line at the mature stage (35 days after the full heading stage). The number of grains per panicle and grain-filling percentage were calculated based on the above data.

### Stress tolerance investigation

The fully filled and uniform rice seeds of the WT and mutants were washed with 70% (v/v) ethanol for 30 s and then washed three times with sterile water. The stress treatment was conducted 3 weeks after sowing. To test chilling tolerance, 27 seedlings per genotype were treated at 2–4 °C for 72 h. Subsequently, they were moved to a temperature-controlled (30 °C day/22 °C night) greenhouse for recovery. For the drought treatment, 27 seedlings per genotype were exposed to dehydration stress in a greenhouse (30 °C day/22 °C night) by withholding water for up to 10 d until variation in the stress response was observed. We then added water for recovery. We compared the salinity tolerance of the WT and homozygous mutant plants in the greenhouse, and the three-week-old plants were treated with fresh groundwater and 0.75% NaCl solution (pH = 7), respectively. After 2 weeks of treatment, the plants were treated with fresh groundwater for recovery. The survival rate was determined after 2 weeks of recovery. Each line was replicated three times.

### Statistical analyses

Statistical analyses were performed using SPSS version 13.0 (SPSS, Chicago, IL, USA) and analyzed with Student’s *t* test and two-way analysis of variance (ANOVA). Values represent the mean ± standard deviation (SD) of three replicates, and significant differences at the 0.05 level are indicated by asterisks or different letters in the figures.

### Yeast two-hybrid analysis

Yeast two-hybrid experiments were performed using the Matchmaker Two-Hybrid System (Clontech). The coding sequences of RGB1, and four PXLGs were amplified using primers listed in additional file 1. Then we cloned the obtained fragments into PGADT7 and PGBKT7 separately. The detail of yeast two hybrid assay was described in elsewhere [31].

## Supplementary information

**Additional file 1.** The primer used in present study

## Data Availability

The datasets used and/or analysed during the current study are available from the corresponding author on reasonable request.

## Abbreviations

CRISPR: Clustered regularly interspaced short palindromic repeats
PXLG: Putative extra-large G protein
QTL: Quantitative trait loci
SD: Standard deviation
WT: Wild type
