# Supplementary material for: Heterotrimeric G protein are involved in the regulation of multiple agronomic traits and stress tolerance in rice
Source: BMC Plant Biol. 2020 Feb 28;20:90. doi: 10.1186/s12870-020-2289-6 (PMC7048073; doi:10.1186/s12870-020-2289-6)
Supplement: Supplementary file 1 — Additional file 1. The primer used in present study [file 12870_2020_2289_MOESM1_ESM.docx]

| Additional file 1. The primer used in present study | | |
| --- | --- | --- |
| Gene | sgRNA | Sequence primer |
| *RGA1* | GTGGATCGATTAGCTGAAGC | GP5064-4033-F:CTGCGTAATAACGGTAGA, GP5064-4033-R:TATTGATGGCAGGTTGGA |
| *RGB1* | GTGATGCAACTGTAAGGCTG | GP3452-2590-F:TGAAGTTGGTGCCCTGTTCT, GP3452-2590-R:CACATGGTAGCAGATGAAGCA |
| *RGG1* | GCAAGAGGCGCGCTTTCTCG | GP3449-2587-F:GAGCCGAGCGAAATGTTAGG, GP3449-2587-R:CGTCTTTTGCTGCTCTGCC |
| *RGG2* | GGCCGCGGCGGAATCATCTG | GP3448-2586-F:CCGCTTCGTCTTCGTCTTCT, GP3448-2586-R:GCCACAGCACAGAGACAGTA |
| *GS3* | GCGGCGCCGCCCGACCCATG | GP5069-3796-F:AGCAAGCCGTACGTGTGTAT, GP5069-3796-R:TAGCTCCGCCATTCAAAGCA |
| *GGC2* | GGCCGCCGCGCTTTCGCTCG | GP5065-4045-F:GCAAATCCGGTCCAAAACCC, GP5065-4045-R:GCTCCCCAATTCCAAATCGC |
| *DEP1* | GCGCGAGATCACGTTCCTCA | GP5548-4436-F:GAGGGGTGGTTCTGAGTTGG, GP5548-4436-R:GGTTTTGAAAAATGCGCGGC |
| *PXLG1* | GCCATCTCATCTGGATTAAG | GP4666-3592-F:ACCCTGTCTGGCTTTTCTCC, GP4666-3592-R:AGGAAGTGGGAGAGCAAGGA |
| *PXLG2* | GGCCTATGACCTACCTAGGG | GP4668-3593-F:ACAACGAGCGGGAATCATCA, GP4668-3593-R:TGTCAGCTCTCCATCCCTCA |
| *PXLG3* | GCATCCCGCTCGACCTGTCG | GP5105-4047-F:CAGCAGCAGCGGAGGAAA, GP5105-4047-R:CGAAGAAAGATCACCCAGTCCA |
| *PXLG4* | GAAGGCATCAAGGTTACCCA | GP5066-4046-F:GATGCTGGACTGGGTTAG, GP5066-4046-R:TTACGCAATGAAGAATGG |
